# Supplementary material for: Highly variable recurrence of tsunamis in the 7,400 years before the 2004 Indian Ocean tsunami
Source: Nat Commun. 2017 Jul 19;8:16019. doi: 10.1038/ncomms16019 (PMC5524937; doi:10.1038/ncomms16019)
Supplement: Supplementary Information [file ncomms16019-s1.pdf]

File name: Supplementary Information
Description: Supplementary Figures, Supplementary Tables and Supplementary References

File name: Peer Review File
Description

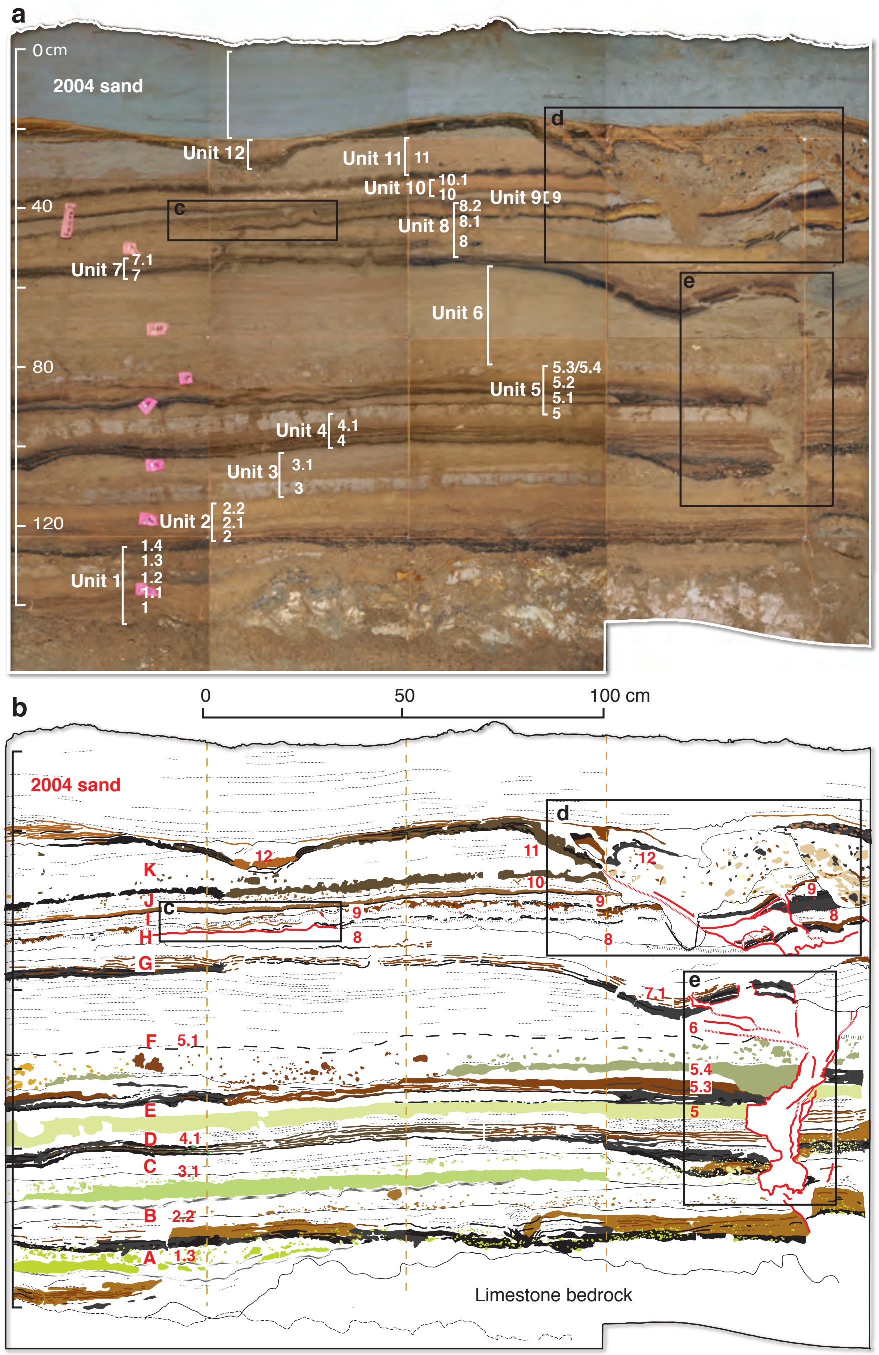

Supplementary Figure 1. Map of Trench 1. (a) Photomosaic of trench 1, face A showing stratigraphic units. **(b)** Trench log, trench 1, face A showing inferred tsunami sand beds and stratigraphic units; selected clayey-mud units are colored; faults colored in red. **(c)** Folding of Unit 8 above flat fault suggests that motion occurred before Unit 9. **(d)** A normal fault offsets Units 8 and 9, about 10 cm. **(e)** High-angle normal fault zone dipping towards the viewer; sense of slip is down on the viewer side, consistent with the a slump away from the wall of the cave. A normal fault offsets units 1-7 and splays upward through Units 6 and 7.

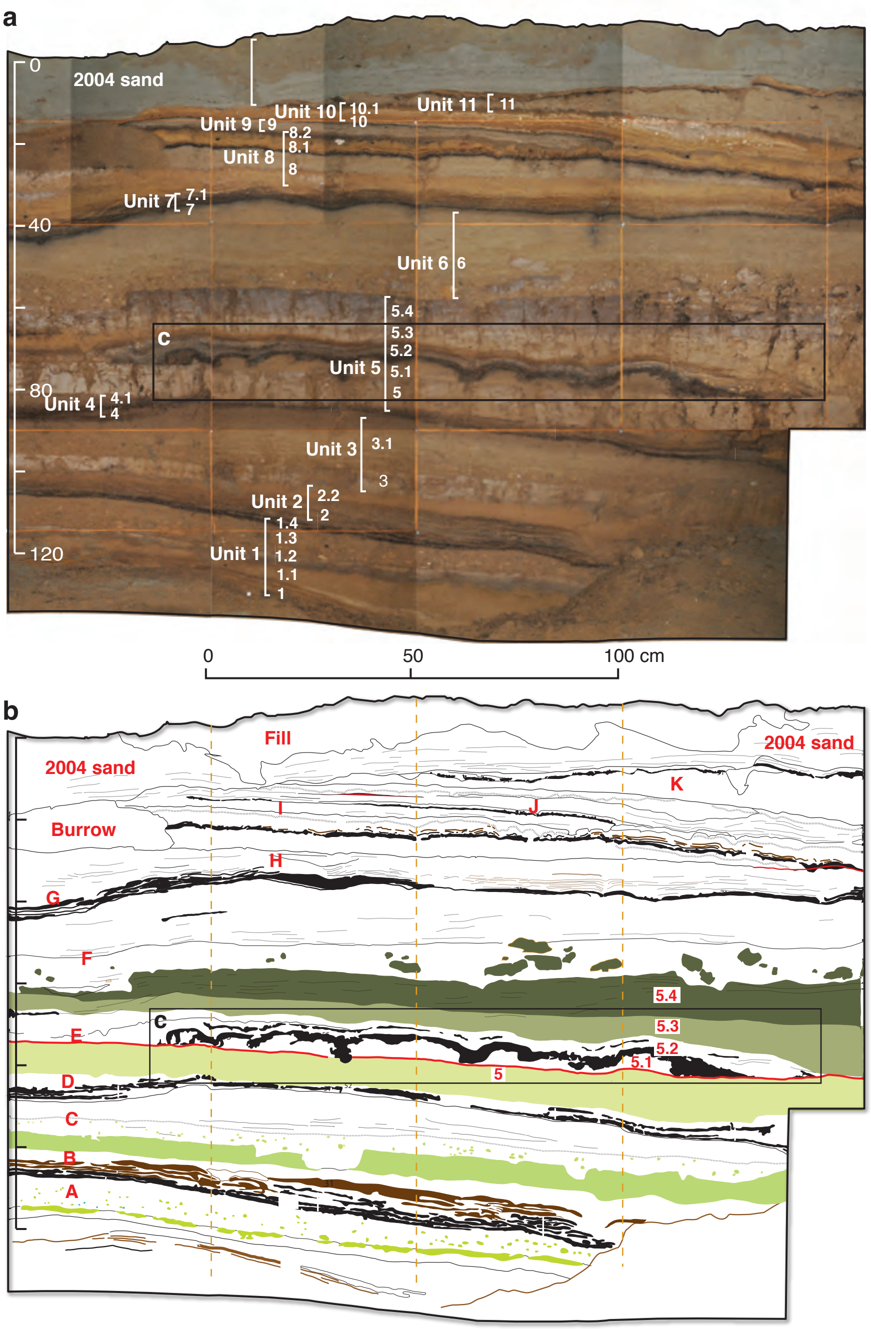

Supplementary Figure 2. Map of Trench 4. (a) Photomosaic of trench 4, face C showing stratigraphic units. **(b)** Trench log, trench 4, face C showing inferred tsunami sand beds; selected clayey-mud units and rip-up clasts are colored; faults colored in red. **(c)** Folding of Units 5.1 and 5.2 and the truncation of the units along the top of Unit 5.3 clayey-mud

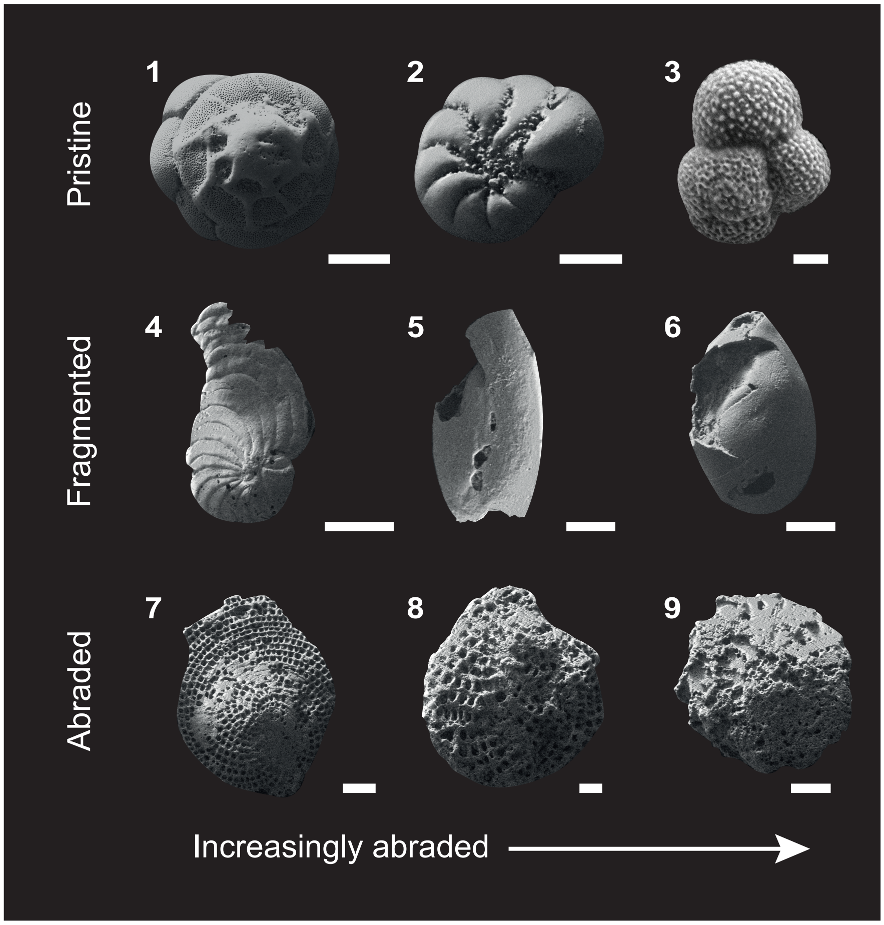

Supplementary Figure 3. Scanning electron microscope images of foraminifera. Scanning electron microscope (SEM) images of taphonomic characters that describe the test condition of individual foraminifera. All scale bars are equal to 100 microns. Numbers 1-3. pristine; 4-6 fragmented; 7-9 abraded individual foraminifera.

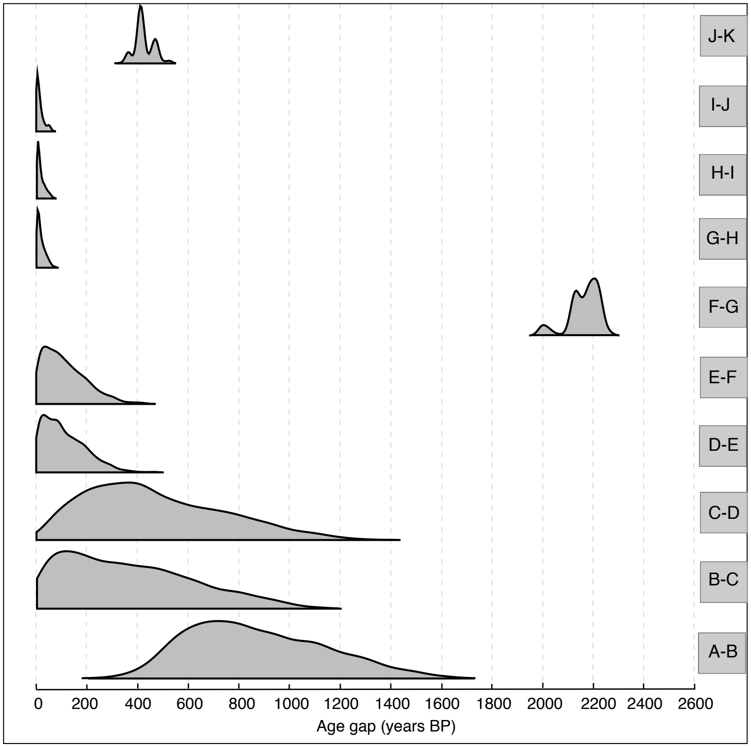

Supplementary Figure 4. Time between consecutive tsunamis. Time between consecutive tsunamis (sand beds A - K) using a custom Bayesian model that simultaneouly calibrates all dates, incorporates the law of superposition and the constraints of limiting dates, which lie between or beyond the range of the directly dated tsunamis. The model is fitted using a Markov chain Monte Carlo approach[1].

Supplementary Table 1. Grain size data for Trench 1.

Unit No.	Depth in unit (cm)	Mean (Φ)	Mode (Φ)	SD (Φ)	% clay (Φ)	% silt (Φ)	% sand (Φ)	Lithologic description	Sed. evidence for tsunamis
13.0	0-1	3.672	3.306	1.163	3.8	22.2	74.0	very fine sand	
13.0	1-2	3.169	3.172	0.485	1.5	5.6	92.9	very fine sand	
13.0	2-3	3.422	3.306	0.898	2.5	15.7	81.8	very fine sand	
13.0	3-4	3.180	3.172	0.477	1.6	5.5	92.8	very fine sand	
13.0	4-5	3.153	3.172	0.419	1.0	4.4	94.5	very fine sand	
13.0	5-6	3.106	3.037	0.439	1.1	4.3	94.5	very fine sand	
13.0	6-7	3.061	3.037	0.461	1.2	4.4	94.4	very fine sand	
13.0	7-8	3.028	3.037	0.586	1.7	5.5	92.8	very fine sand	
13.0	8-9	2.978	2.902	0.912	2.3	10.7	87.0	fine sand	
13.0	9-10	2.854	2.902	0.542	1.2	3.9	94.9	fine sand	
13.0	10-11	2.888	2.902	0.608	1.5	4.9	93.5	fine sand	
13.0	11-12	2.876	2.902	0.635	1.6	5.2	93.2	fine sand	
13.0	12-13	2.790	2.902	0.891	2.0	6.2	91.8	fine sand	
13.0	13-14	2.758	2.902	0.739	1.2	3.8	94.9	fine sand	
13.0	14-15	2.776	2.902	1.261	3.2	8.2	88.6	fine sand	
13.0	15-16	2.774	2.902	0.903	1.7	6.0	92.4	fine sand	
13.0	16-17	2.763	2.902	0.796	1.3	4.8	93.9	fine sand	
13.0	17-18	2.773	2.902	0.907	1.7	6.2	92.1	fine sand	
13.0	18-19	2.701	2.768	0.631	0.9	2.6	96.6	fine sand	
13.0	19-20	2.687	2.768	0.718	1.2	3.8	94.9	fine sand	
13.0	20-21	2.875	2.902	0.767	2.2	6.0	91.8	fine sand	
13.0	21-22	2.719	2.768	0.615	1.1	3.6	95.3	fine sand	Fining upward
13.0	22-23	2.473	2.230	0.699	1.0	2.6	96.4	fine sand	
13.0	23-24	2.463	2.230	0.727	1.0	2.8	96.2	fine sand	Coarse pulse
13.0	24-25	2.690	2.768	0.794	1.8	5.7	92.5	fine sand	
13.0	25-26	2.549	2.768	0.817	1.3	4.5	94.2	fine sand	
13.0	26-27	2.570	2.902	0.740	0.9	2.9	96.2	fine sand	
13.0	27-28	2.641	2.902	0.742	0.9	3.3	95.8	fine sand	
13.0	28-29	2.665	2.902	0.872	1.5	5.4	93.1	fine sand	
13.0	29-30	2.742	2.902	1.026	2.2	6.8	91.1	fine sand	
13.0	30-31	2.588	2.768	1.065	1.9	7.2	90.9	fine sand	Fining upward
13.0	31-32	2.244	2.230	0.867	1.2	3.4	95.4	fine sand	
13.0	32-33	2.300	2.364	0.883	1.0	3.3	95.7	fine sand	Coarse pulse
13.0	33-34	2.665	2.902	1.072	2.3	7.1	90.6	fine sand	
13.0	34-35	2.628	2.768	0.959	1.6	6.8	91.6	fine sand	
13.0	35-36	2.688	2.902	1.113	2.2	8.8	89.0	fine sand	
13.0	36-37	2.549	2.768	0.950	1.8	5.4	92.8	fine sand	
13.0	37-38	2.695	2.902	1.230	2.5	10.1	87.4	fine sand	
13.0	38-39	2.709	2.902	1.270	2.7	10.2	87.0	fine sand	
13.0	39-40	2.465	2.095	1.341	3.0	8.7	88.3	fine sand	Fining upward
13.0	40-41	2.246	2.095	1.352	2.5	7.9	89.7	fine sand	
13.0	41-42	2.084	1.960	1.019	1.9	4.1	94.1	fine sand	
13.0	42-43	2.376	2.095	1.544	3.6	10.5	85.9	fine sand	Coarse pulse

Supplementary Table 1. Continued

Unit No.	Depth in unit (cm)	Mean (Φ)	Mode (Φ)	SD (Φ)	% clay (Φ)	% silt (Φ)	% sand (Φ)	Lithologic description	Sed. evidence for tsunamis
11.0	0-1	2.428	2.364	0.772	1.3	4.4	94.3	fine sand	
11.0	1-2	2.415	2.364	0.828	1.2	5.0	93.8	fine sand	
11.0	2-3	2.443	2.364	0.755	1.2	4.5	94.3	fine sand	
11.0	3-4	2.493	2.364	0.723	1.2	4.6	94.2	fine sand	
11.0	4-5	2.492	2.364	1.031	1.9	8.4	89.7	fine sand	
11.0	5-6	2.561	2.230	1.137	2.1	10.5	87.3	fine sand	
11.0	6-7	2.473	2.230	1.274	2.7	10.9	86.4	fine sand	
11.0	7-8	2.204	2.230	0.796	1.5	3.9	94.6	fine sand	
11.0	8-9	2.254	2.095	1.265	2.9	8.2	88.9	fine sand	
11.0	9-10	2.178	2.095	1.063	1.9	5.3	92.8	fine sand	
11.0	10-11	2.734	2.230	1.683	3.6	14.4	82.0	fine sand	
11.0	11-12	2.030	1.960	1.312	2.3	8.3	89.4	fine sand	
11.0	12-13	1.856	2.095	0.971	1.1	3.9	95.1	medium sand	
11.0	13-14	1.887	2.095	0.937	1.0	3.5	95.5	medium sand	Fining upward
10.2	14-15	2.174	2.230	1.173	1.8	7.2	91.1	fine sand	
10.2	15-16	2.795	2.364	1.695	4.0	13.6	82.4	fine sand	
10.2	16-17	2.537	2.364	1.021	2.0	8.0	90.0	fine sand	
6.0	0-1	2.945	2.499	1.353	3.8	12.8	83.3	fine sand	
6.0	1-2	3.456	2.499	1.753	5.2	15.7	79.1	very fine sand	
6.0	2-3	2.631	2.499	0.664	1.7	4.8	93.4	fine sand	
6.0	3-4	2.646	2.499	0.868	2.4	6.1	91.5	fine sand	
6.0	4-5	2.617	2.499	0.991	2.0	7.7	90.2	fine sand	
6.0	5-6	2.587	2.364	1.020	2.8	7.0	90.2	fine sand	
6.0	6-7	2.593	2.364	1.103	3.3	7.7	89.0	fine sand	
6.0	7-8	2.519	2.364	1.204	2.3	9.9	87.8	fine sand	
6.0	8-9	2.336	2.230	0.910	1.9	4.9	93.2	fine sand	
6.0	9-10	2.429	2.230	1.235	2.9	8.3	88.9	fine sand	
6.0	10-11	2.328	2.230	0.904	1.7	5.2	93.1	fine sand	
6.0	11-12	2.423	2.364	1.145	2.7	7.1	90.2	fine sand	
6.0	12-13	3.463	2.230	2.420	8.9	16.4	74.8	very fine sand	
6.0	13-14	2.327	2.230	1.528	4.1	9.7	86.2	fine sand	
6.0	14-15	2.196	2.095	1.300	2.8	7.9	89.3	fine sand	
6.0	15-16	3.184	2.095	2.162	5.3	17.9	76.7	very fine sand	
6.0	16-17	2.388	2.230	1.699	3.7	11.8	84.5	fine sand	
6.0	17-18	2.831	2.095	2.172	5.4	16.0	78.5	fine sand	
6.0	18-19	2.645	2.230	1.983	4.3	15.6	80.0	fine sand	
6.0	19-20	2.980	2.230	1.958	5.3	17.9	76.8	fine sand	
6.0	20-21	3.218	2.230	2.168	6.2	21.3	72.6	very fine sand	
6.0	21-22	4.823	2.364	2.686	15.5	40.9	43.7	silt	
6.0	22-23	2.851	2.230	2.038	5.8	15.1	79.2	fine sand	
6.0	23-24	3.851	2.230	2.911	13.0	24.7	62.4	very fine sand	
6.0	24-25	2.578	2.364	1.107	2.4	9.4	88.2	fine sand	
6.0	25-26	2.506	2.230	1.023	2.1	8.4	89.5	fine sand	
6.0	26-27	2.313	2.230	1.159	1.8	7.8	90.5	fine sand	
6.0	27-28	2.078	2.095	0.898	1.4	4.2	94.4	fine sand	
6.0	28-29	2.370	2.095	1.484	2.8	11.7	85.5	fine sand	

Supplementary Table 1. Continued

Unit No.	Depth in unit (cm)	Mean (Φ)	Mode (Φ)	SD (Φ)	% clay (Φ)	% silt (Φ)	% sand (Φ)	Lithologic description	Sed. evidence for tsunamis
5.5	n/a	n/a	n/a	n/a	n/a	n/a	n/a	n/a	
5.4	n/a	n/a	n/a	n/a	n/a	n/a	n/a	n/a	
5.3	n/a	n/a	n/a	n/a	n/a	n/a	n/a	n/a	
5.2	0-1	2.982	2.230	1.926	4.7	20.6	74.7	fine sand	
5.2	1-2	4.380	2.364	2.378	10.3	36.3	53.5	silt	
5.2	2-3	4.669	2.364	2.471	12.1	41.1	46.7	silt	
5.1	3-4	4.950	2.499	2.446	15.4	41.8	42.8	silt	
5.1	4-5	2.688	2.499	0.933	2.2	9.4	88.4	fine sand	
5.1	5-6	2.727	2.364	1.173	2.5	12.4	85.2	fine sand	
5.1	6-7	3.075	2.230	2.051	5.7	18.9	75.4	very fine sand	
5.0	n/a	n/a	n/a	n/a	n/a	n/a	n/a	n/a	
4.1	7-8	2.487	2.230	1.311	2.5	10.6	86.8	fine sand	
4.0	8-9	2.320	2.230	1.119	1.8	7.9	90.2	fine sand	
3.2	0-1	2.548	2.095	1.474	2.9	12.8	84.3	fine sand	
3.2	1-2	2.228	2.095	1.059	1.9	7.1	91.1	fine sand	
3.2	2-3	2.148	2.095	1.051	1.9	5.7	92.4	fine sand	
3.2	3-4	3.266	2.230	2.215	6.1	20.6	73.3	very fine sand	
3.2	4-5	2.082	1.960	1.349	2.1	9.5	88.4	fine sand	
3.1	5-6	1.718	1.691	1.428	2.0	7.9	90.1	medium sand	
3.1	6-7	2.294	1.960	1.608	2.8	12.3	84.9	fine sand	
2.0	7-8	3.773	2.095	2.952	10.8	31.3	57.9	very fine sand	
2.0	8-9	4.586	2.230	2.541	13.1	38.2	48.7	silt	
2.2	1-2	4.399	2.633	2.248	11.6	30.7	57.6	silt	
2.2	2-3	2.902	2.633	1.105	3.2	11.6	85.2	fine sand	
2.2	3-4	3.078	2.499	1.225	2.5	14.9	82.6	very fine sand	
2.1	4-5	2.616	2.364	0.881	1.7	8.2	90.1	fine sand	
2.1	5-6	2.536	2.364	1.107	1.8	9.6	88.6	fine sand	
2.1	6-7	2.368	2.230	1.149	1.8	8.3	90.0	fine sand	
2.0	7-8	2.711	2.230	1.528	3.2	13.2	83.6	fine sand	
2.0	8-9	2.437	2.230	1.280	2.8	8.9	88.3	fine sand	
2.0	9-10	2.607	2.230	1.617	3.3	13.2	83.6	fine sand	
1.4	0-1	2.341	2.095	1.641	3.4	11.6	85.0	fine sand	
1.3	1-2	2.674	2.230	1.858	4.1	15.2	80.8	fine sand	
1.3	2-3	3.090	2.230	2.153	4.8	22.3	73.0	very fine sand	
1.3	3-4	2.976	2.230	2.115	4.9	21.9	73.3	fine sand	
1.3	4-5	3.165	2.230	2.231	5.8	24.4	69.8	very fine sand	
1.3	5-6	2.795	2.230	2.010	3.9	18.6	77.6	fine sand	
1.2	n/a	n/a	n/a	n/a	n/a	n/a	n/a	n/a	
1.1	6-7	3.061	2.364	1.830	4.7	18.5	76.8	very fine sand	
1.1	7-8	4.330	2.902	1.938	7.4	40.7	51.9	silt	
1.1	8-9	3.514	2.364	1.950	5.5	24.8	69.7	very fine sand	
1.1	9-10	3.401	2.230	1.932	4.1	23.5	72.4	very fine sand	

Supplementary Table 1. Continued

Unit No.	Depth in unit (cm)	Mean (Φ)	Mode (Φ)	SD (Φ)	% clay (Φ)	% silt (Φ)	% sand (Φ)	Lithologic description	Sed. evidence for tsunamis
1.1	10-11	2.490	2.230	1.036	2.1	8.2	89.8	fine sand	
1.1	11-12	2.506	2.230	1.255	2.4	9.8	87.8	fine sand	
1.1	12-13	2.401	2.230	1.192	2.0	9.0	88.9	fine sand	
1.1	13-14	2.616	2.230	1.701	2.5	15.2	82.3	fine sand	
1.0	n/a	n/a	n/a	n/a	n/a	n/a	n/a	n/a	

Supplementary Table 1. Grain size data for Trench 1. Grain size data, Trench 1, face A. Mean, mode, standard deviation (SD), % clay, % silt, and % sand values are indicated. Lithologic descriptions are based on mean grain size values, and described using the Wentworth-Phi Scale[2]. Intervals for which grain size data are not available are indicated by n/a.

Supplementary Table 2. Grain size data for Trench 4.

Unit No.	Depth in unit (cm)	Mean (Φ)	Mode (Φ)	SD (Φ)	% clay (Φ)	% silt (Φ)	% sand (Φ)	Lithologic description	Sed. evidence for tsunamis
10.1	3-4	2.631	2.521	1.141	2.3	8.6	89.1	fine sand	
10.1	4-5	2.983	2.336	1.560	3.6	13.6	82.7	fine sand	
10.0	5-6	3.510	2.336	2.113	6.1	23.6	70.4	very fine sand	
10.0	6-7	2.884	2.336	1.789	4.2	15.0	80.9	fine sand	
9.1	n/a	n/a	n/a	n/a	n/a	n/a	n/a	n/a	
9.0	7-8	2.340	2.152	1.571	3.5	10.6	86.0	fine sand	
9.0	8-9	2.258	2.336	1.624	2.8	10.5	86.7	fine sand	
8.3	9-10	5.754	5.283	2.033	13.0	70.8	16.1	silt	
8.3	10-11	5.665	6.204	2.342	16.8	59.6	23.6	silt	
8.2	11-12	3.859	2.889	1.944	7.6	14.9	77.5	very fine sand	
8.2	12-13	3.407	2.705	1.640	4.4	16.5	79.1	very fine sand	
8.2	13-14	3.279	2.521	1.579	4.1	15.8	80.2	very fine sand	
8.2	14-15	2.753	2.521	1.241	4.0	9.0	87.1	fine sand	
8.2	15-16	2.591	2.521	1.104	2.6	6.7	90.7	fine sand	
8.1b	16-17	2.432	2.336	1.023	1.7	5.6	92.7	fine sand	
8.1a	17-18	2.301	2.336	0.733	0.8	2.3	96.8	fine sand	
8.1a	18-19	2.253	2.336	0.997	1.5	3.9	94.5	fine sand	
8.1a	19-20	2.328	2.336	1.127	2.1	6.1	91.8	fine sand	
8.1a	20-21	2.220	2.336	1.080	1.9	4.7	93.4	fine sand	
8.1	21-22	4.604	2.521	2.575	12.5	41.0	46.5	silt	
8.1	22-23	4.812	2.521	2.676	14.9	40.2	45.0	silt	
8.1	23-24	4.518	2.521	2.778	16.8	29.0	54.3	silt	
8.1	24-25	4.723	2.521	3.037	21.5	25.5	53.1	silt	
8.1	25-26	4.189	2.336	2.838	15.7	17.2	67.1	silt	
8.1	26-27	4.466	2.705	2.869	16.4	28.7	55.0	silt	
8.0	27-28	3.823	2.705	2.014	8.7	19.6	71.7	very fine sand	
8.0	28-29	3.096	2.705	1.503	5.3	11.9	82.8	very fine sand	
8.0	29-30	3.330	2.521	1.821	6.7	13.1	80.2	very fine sand	
8.0	30-31	2.469	2.336	1.282	3.7	6.8	89.5	fine sand	
8.0	31-32	2.933	2.336	1.809	5.3	13.1	81.6	fine sand	
8.0	32-33	2.416	2.336	1.698	4.7	10.1	85.3	fine sand	
8.0	33-34	2.059	2.152	1.778	3.6	9.2	87.2	fine sand	
8.0	34-35	1.941	2.152	1.572	2.3	7.0	90.7	medium sand	
8.0	35-36	2.064	2.336	1.788	3.5	9.2	87.4	fine sand	
8.0	36-37	2.303	2.152	2.189	4.6	13.1	82.3	fine sand	Fining upward
7.2	37-38	3.056	2.336	2.573	7.6	19.5	72.9	very fine sand	
7.2	38-39	5.181	2.521	2.845	19.6	40.7	39.8	silt	
7.2	39-40	5.093	2.705	2.739	18.4	38.2	43.4	silt	
7.2	40-41	4.876	2.705	2.556	15.0	39.0	46.1	silt	
7.2	41-42	4.568	2.705	2.414	13.1	35.7	51.2	silt	Fining upward

Supplementary Table 2. Continued

Unit No.	Depth in unit (cm)	Mean (Φ)	Mode (Φ)	SD (Φ)	% clay (Φ)	% silt (Φ)	% sand (Φ)	Lithologic description
7.1	42-43	3.653	2.705	2.155	7.9	19.3	72.8	very fine sand
7.1	43-44	2.989	2.705	2.210	5.9	16.4	77.7	fine sand
7.1	44-45	2.594	2.705	1.814	4.1	12.3	83.7	fine sand
7.1	45-46	2.595	2.705	1.657	3.6	11.3	85.1	fine sand
7.0	46-47	2.654	2.705	1.530	3.1	10.7	86.2	fine sand
7.0	47-48	2.522	2.521	1.178	1.9	5.5	92.6	fine sand

Supplementary Table 2. Grain size data for Trench 4. Grain size data for trench 4, face D (location shown on Fig 2). Mean, mode, standard deviation (SD), % clay, % silt, and % sand values are indicated. Lithologic descriptions are based on mean grain size values and described using the Wentworth-Phi Scale[2]. Intervals for which grain size data are not available are indicated by n/a.

			Foraminiferal taxonomy			Foraminiferal taphonomy			
Unit No.	Depth in unit (cm)	Total con. per 1 cm3	% Intertidal species	% Subtidal species	% Planktic species	Pristine (%)	Abraded (%)	Frag'ed (%)	Cave chalk taphonomy
13.0	2-3	2500	38	54	8	41	34	25	weathered
13.0	20-21	2799	34	52	14	51	32	17	weathered
13.0	35-36	3246	33	58	9	52	26	22	weathered
12.0	n/a	n/a	n/a	n/a	n/a	n/a	n/a	n/a	n/a
11.0	6-7	2485	33	56	11	55	31	14	weathered
11.0	7-8	1976	30	61	9	52	29	19	weathered
11.0	11-12	2355	38	48	14	49	33	18	weathered
11.0	13-14	2028	26	60	14	52	26	22	weathered
10.2	n/a	n/a	n/a	n/a	n/a	n/a	n/a	n/a	n/a
6.0	2-3	229	36	55	9	69	21	10	none
6.0	8-9	198	18	68	14	65	26	9	none
6.0	14-15	296	30	57	13	51	30	19	none
6.0	18-19	201	53	43	4	48	46	6	weathered
6.0	26-27	224	35	51	14	41	49	10	weathered
5.5	n/a	n/a	n/a	n/a	n/a	n/a	n/a	n/a	n/a
5.4	1-2	0	0	0	0	0	0	0	none
5.3	1-2	2	100	0	0	0	100	0	pristine
5.2	2-3	0	0	0	0	0	0	0	none
5.1	4-5	35	56	44	0	28	63	9	none
5.1	5-6	42	62	37	1	20	66	14	none
5.0	0-1	0	0	0	0	0	0	0	pristine
4.1	7-8	19	83	17	0	0	78	22	none
4.0	8-9	0	0	0	0	0	0	0	none
3.2	1-2	328	45	46	9	33	32	35	none
3.2	3-4	412	40	53	7	32	46	22	weathered
3.1	5-6	241	42	52	6	41	31	28	weathered
3.1	6-7	146	37	61	2	37	44	19	weathered
3.0	7-8	32	46	54	0	45	43	12	pristine
3.0	8-9	55	51	49	0	43	51	6	none
2.2	2-3	89	51	47	2	9	44	47	weathered
2.2	3-4	76	59	41	0	12	33	55	weathered
2.1	5-6	68	55	45	0	8	58	34	pristine
2.0	8-9	94	62	38	0	20	49	31	pristine
2.0	9-10	83	66	34	0	14	53	33	pristine
1.4	n/a	n/a	n/a	n/a	n/a	n/a	n/a	n/a	n/a
1.3	1-2	34	41	58	1	2	68	30	none
1.3	2-3	62	43	57	0	9	77	14	none
1.3	3-4	21	62	38	0	5	73	22	none
1.2	0-1	20	71	29	0	0	52	48	none
1.1	7-8	0	0	0	0	0	0	0	none
1.1	10-11	0	0	0	0	0	0	0	none
1.1	13-14	0	0	0	0	0	0	0	none
1.0	n/a	n/a	n/a	n/a	n/a	n/a	n/a	n/a	n/a

Supplementary Table 3. Formaninfera and Cave Calk Analyses, Trench 1, Face A. Foraminiferal taxonomy and taphonomy, and cave chalk data for trench 1, face A (Units 1 – 12). Intervals for which foraminiferal and cave chalk data are not available are indicated by n/a. Frag'ed, fragmented.

Unit No.	Depth in unit (cm)	Total con. per 1 cm3	Foraminiferal taxonomy % Intertidal species	% Subtidal species	% Planktic species	Foraminiferal taphonomy Pristine (%)	Abraded (%)	Frag'ed (%)	Cave chalk taphonomy
10.1	4-5	190	45	51	4	53	36	11	weathered
10.0	5-6	9	86	14	0	0	24	76	pristine
9.1	0-1	4	54	46	0	8	82	10	pristine
9.0	8-9	256	33	58	9	44	40	16	weathered
8.3	9-10	20	55	45	0	0	96	4	pristine
8.2	13-14	0	0	0	0	0	0	0	none
8.1b	16-17	93	68	28	4	14	34	52	weathered/pristine
8.1a	18-19	0	0	0	0	0	0	0	pristine
8.1	n/a	n/a	n/a	n/a	n/a	n/a	n/a	n/a	n/a
8.0	30-31	62	0	100	0	39	45	16	weathered
8.0	34-35	55	2	98	0	44	53	3	weathered
7.2	22-23	0	0	0	0	0	0	0	none
7.1	42-43	105	17	77	6	65	24	11	weathered
7.1	44-45	93	18	71	11	82	12	6	weathered
7.0	n/a	n/a	n/a	n/a	n/a	n/a	n/a	n/a	n/a

Supplementary Table 4. Formaninfera and Cave Calk Analyses, Trench 4, Face D. Foraminiferal taxonomy and taphonomy, and cave chalk data for trench 4, face D (location shown in Fig. 2). Intervals for which foraminiferal and cave chalk data are not available are indicated by n/a.

Stratigraphic Unit	Tsunami event #	Thickness (cm)	Standard deviation	Standard error
12	2004	21.2	3.676	1.108
11	K	8.4	4.195	1.326
10.1	J	4.2	1.047	0.24
9	I	5.3	4.921	1.073
8	H	7.3	3.5	0.7
7.1	G	2.4	0.868	0.17
6	F	23.2	6.1	1.1
5.1	E	2.8	1.039	0.259
4.1	D	2.7	1.203	0.245
3.1/3.2	C	9.2	3	0.6
2.2	B	7.4	2.358	0.471
1.3	A	5.4	1.734	0.447

Supplementary Table 5. Tsunami Sand Bed thickness. Sand bed (A - K) thickness, including the 2004 tsunami deposit. Events are listed in stratigraphic order. *The stratigraphic thickness is the average across 29 vertical sections in Trench 1 Faces A and C (Supplementary Figures S1 and S2).

Sand Layer	Regression limits*								
	1%	2.50%	5%	25%	50%	75%	95%	97.50%	99%
K	2803	2807	2815	2857	2866	2875	2916	2919	2925
J	3264	3268	3270	3275	3280	3326	3341	3343	3349
I	3271	3275	3278	3288	3299	3333	3346	3349	3354
H	3278	3284	3287	3304	3327	3341	3353	3356	3358
G	3290	3297	3304	3332	3344	3353	3363	3369	3375
F	5337	5343	5357	5475	5519	5549	5575	5578	5580
E	5387	5428	5480	5557	5614	5680	5770	5801	5834
D	5515	5544	5578	5671	5734	5791	5866	5880	5896
C	5783	5816	5857	5986	6156	6369	6680	6756	6821
B	5918	6001	6083	6384	6598	6769	6915	6951	6989
A	7230	7286	7324	7401	7436	7469	7529	7539	7548

Supplementary Table 6. Confidence Interval Limits for the Age of each Sand Bed (A - K).
*Bayesian age-depth model that fits 19 radiocarbon dates, some of which directly date a tsunami while others lie in between or provide maximum age limits for a subset of the tsunamis

Sand Bed	Regression limits								
	1%	2.50%	5%	25%	50%	75%	95%	97.50%	99%
J_K	352	357	364	406	418	457	486	505	526
I_J	0	0	1	4	10	20	50	55	59
H-I	0	0	1	4	10	23	47	53	59
G_H	0	0	1	6	13	26	49	56	65
F-G	1988	1997	2013	2131	2178	2210	2238	2247	2264
E_F	2	4	8	45	97	165	276	310	365
D_E	1	3	7	42	92	164	272	301	351
C_D	48	72	96	260	420	656	962	1057	1130
B_C	4	15	28	148	326	539	844	917	990
A_B	387	446	491	660	832	1063	1358	1453	1519

Suppleme mtary Table 7. Confidence interval limits for the age gap between each tsunami.

Species ecology	Dominant taxa
Shallow intertidal species:	*Ammonia convexa* Collins, 1958
	Ammonia parkinsoniana (d'Orbigny, 1839)
	Ammonia tepida (Cushman, 1926)
	Calcarina spp.
	Elphidium advenum (Cushman, 1922)
	Elphidium craticulatum (Fichtel and Moll, 1798)
	Elphidium crispum (Linnaeus, 1758)
	Elphidium excavatum (Terquem, 1875)
	Elphidium spp.
	Peneroplis proteus (d'Orbigny, 1839)
	Miliolids
Deeper subtidal species:	*Asterorotalia* sp.
	Amphistegina lessonii d'Orbigny in Guerin-Meneville, 1843
	Amphistegina radiata (Fichtel and Moll, 1798)
	Bolivina spp.
	Cibicides lobatulus (Walker and Jacob, 1798)
	Cibicides refulgens de Montfort, 1808
	Cibicides spp.
	Epinoides repandus (Fichtel and Moll, 1798)
	Heterolepa sp.
	Lagena sp.
	Operculina ammonoides Sidebottom, 1918
	Pararotalia sp.
	Pararotalia stellata (de Férussac, 1827)
Offshore species:	Planktics

Supplememtary Table 8. Dominant foraminifera contained within cave sediments

Supplementary References

1. Brooks, S., Gelman, A., Jones, G., Meng, X.L. (eds) *Handbook of Markov Chain Monte Carlo*. CRC Press (2011).

2. Blott, S. J. & Pye, K. Gradistat: a grain size distribution and statistics package for the analysis of unconsolidated sediments. *Earth Surf. Proc. Land.* **26,** 1237-1248 (2001).
